# A novel *C19ORF12* mutation in two MPAN sisters treated with deferiprone

**DOI:** 10.1186/s12883-023-03172-z

**Published:** 2023-03-31

**Authors:** Sihui Chen, Xiaohui Lai, Jiajia Fu, Jing Yang, Bi Zhao, Huifang Shang, Rui Huang, Xueping Chen

**Affiliations:** Department of Neurology, West China Hospital of Sichuan University, Chengdu, Sichuan Province China

**Keywords:** *C19orf12*, Mitochondrial membrane protein-associated neurodegeneration, MPAN, NBIA, Deferiprone, DFP

## Abstract

**Background:**

Mitochondrial membrane protein-associated neurodegeneration (MPAN) is a rare and devastating disease caused by pathogenic mutations in *C19orf12* gene. MPAN is characterized by pathological iron accumulation in the brain and fewer than 100 cases of MPAN have been described. Although the diagnosis of MPAN has achieved a great breakthrough with the application of the whole exome gene sequencing technology, the therapeutic effect of iron chelation therapy in MPAN remains controversial.

**Case presentation:**

We reported that two sisters from the same family diagnosed with MPAN had dramatically different responses to deferiprone (DFP) treatment. The diagnosis of MPAN were established based on typical clinical manifestations, physical examination, brain magnetic resonance imaging (MRI), cerebrospinal fluid analysis (CSF) and gene sequencing results. The clinical presentations of the two sisters with MPAN due to novel gene locus mutations were similar to those previously reported. There is no other difference in basic information except that the proband had a later onset age and fertility history. Both the proband and his second sister were treated with deferiprone (DFP), but they had dramatically different responses to the treatment. The proband’s condition deteriorated sharply after treatment with DFP including psychiatric symptoms and movement disorders. However, the second sister of the proband became relatively stable after receiving the DFP treatment. After four years of follow-up, the patient still denies any new symptoms of neurological deficits.

**Conclusion:**

The findings of this study enriched the MPAN gene database and indicated that DFP might ameliorate symptom progression in patients without severe autonomic neuropsychiatric impairment at the early stage of the disease.

## Introduction

Neurodegeneration with Brain Iron Accumulation (NBIA) is characterized by excessive brain iron deposition, especially in the globus pallidus (GP) and substantia nigra (SN) in the basal ganglia area [1, 2]. The most common type is Pantothenase kinase-associated neurodegeneration (PKAN), which accounts for approximately 50% of NBIA. Mitochondrial Membrane Protein-Associated Neurodegeneration (MPAN) caused by mutations in *C19orf12 (open reading frame 12 on chromosome 19)* is the other subtype of NBIA [2–5]. The clinical symptoms of MPAN mainly affect the pyramidal and extrapyramidal systems, and the patients can present various manifestations during progression, including gait disorder and clumsiness, dystonia, ataxia, swallowing disorder, neuropsychiatric abnormalities, cognitive disabilities, and optic atrophy. At the end of the disease stage, severe dementia, urinary incontinence are frequently presented [1, 5–7]. Patients with MPAN have imaging features of hyperintense streaks in the medial medullary plate on Magnetic resonance image (MRI) T2 weighted, and axonal motor neuropathy is a late feature [8]. At present, the diagnostic clues for MPAN include clinical manifestations, MRI features, and sequence analysis of *C19orf12*. However, there is still a lack of effective treatment for MPAN. DFP, with a low molecular weight, favorable octanol-water partition coefficient and lipophilicity, it has proved that it can cross the blood-brain barrier to play its role of chelating iron ions and has been attempt to treat patients with NBIA [8].

## Case presentation

**Fig. 1:**
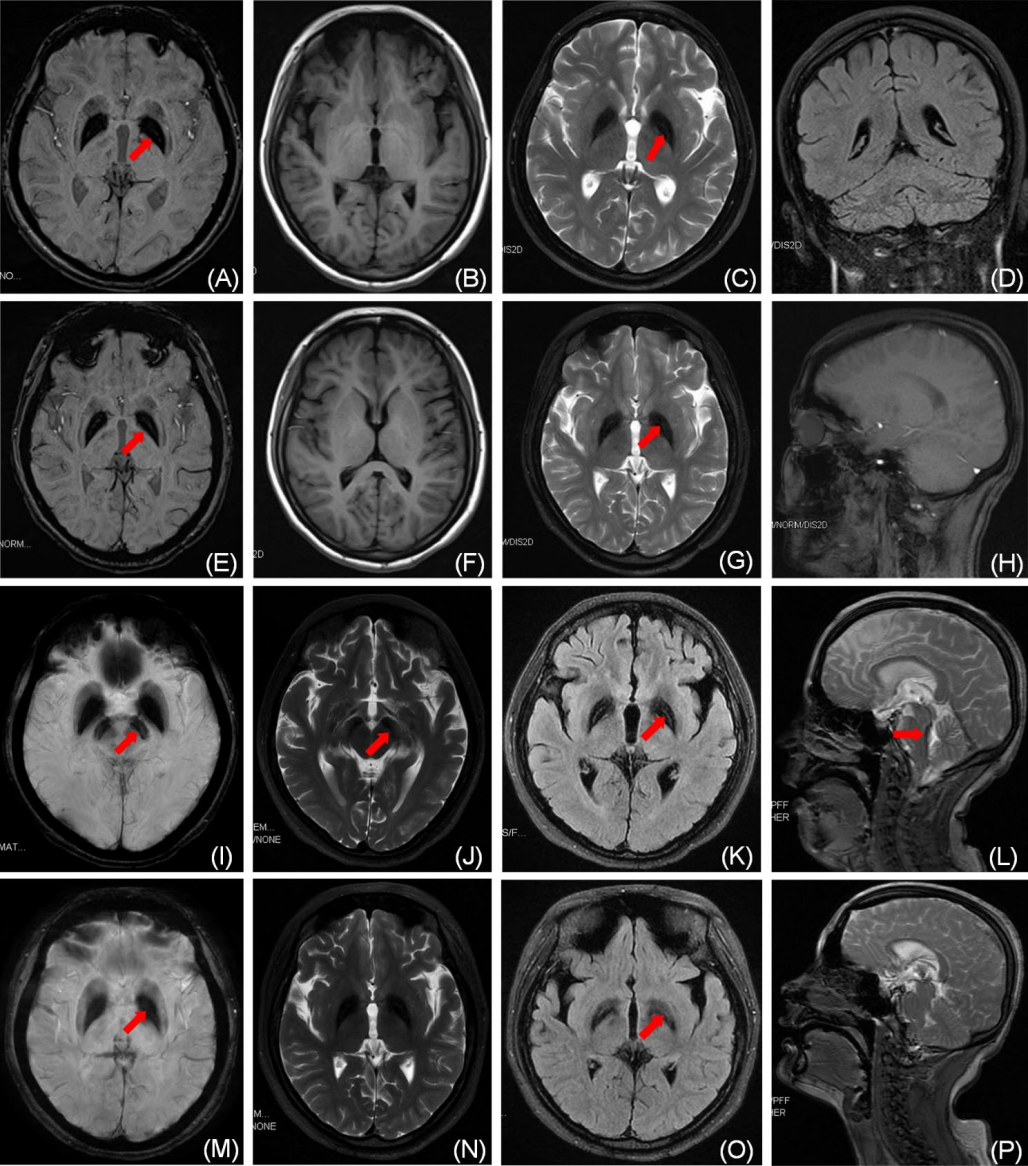
Nuclear Magnetic Resonance Imaging (A-D) show the MRI images of proband in 2014. (E-H) Shows MRI images of the proband ‘s sister at the hospital in 2014. (I-L) The MRI images of the proband in 2017; Symmetric hypodense lesions of GP and SN in T2-weighted (I) and SWI images (K) of brain imaging. (M-P) The images of her sister when she was hospitalized in 2017. The linear high signal is visible in the endomyeloid plate between the medial and lateral low signal without “tiger eye“(red arrow in figure A, C,E,G,I-L,M,O).GP, globus, pallidum; SN, substantia nigra

The proband was a 31-year-old female with a negative family history, and she was the first child of a consanguineous marriage. Her parents were cousins. Her first neurological symptoms, which developed at the age of 24 years, were gait impairment. Stiffness in both lower limbs and gait difficulties led to frequent falls. The clinical symptoms of the proband in this study before pregnancy were actually lighter than her second sister. At the age of 25 years, the patient showed increased irritability, concentration disturbances and memory problems. At the age of 26 years, the patient showed mild dysarthria and dysphagia. These symptoms of this patient gradually worsened until she was pregnant at the age of 28 years, and her condition deteriorated rapidly. In just a few months, she showed a spastic-dystonic gait pattern, bilateral pes cavus, and inability to walk independently. Stiffness of upper extremities, generalized dystonia, and postural instability was also present. After the patient gave birth, her symptoms still did not improve and continued to worsen as progressively as during pregnancy. On admission in 2014, she was bedridden. Cognitive impairment, indifference, and urinary and fecal incontinence were also present. The neurological examination revealed speaking and swallowing disturbances, chorea in the upper limbs, brisk deep tendon reflexes, rigidity and spasticity of extremities, and bilateral Babinski sign positive. Ophthalmological investigation did not show retinopathy or optic atrophy. Laboratory tests including liver and kidney work, electrolytes, blood routine, myocardial markers, brain natural peptide (BNP), glycated hemoglobin, coagulation function, inflammatory indicators, homocysteine levels, tumor markers, immune function, the plasma levels of iron, ceruloplasmin, ferritin, transferrin and stool routine examination showed no obvious abnormalities. It was noted that when the proband was hospitalized, the examination revealed significant urinalysis abnormalities, occult blood, suspected positive urine protein, bacterial count > 1500/UL, which was thought to be related to her long-term urinary incontinence. The surface electromyography (EMG) showed normal CMAP amplitudes and distal motor latencies. The motor conduction velocities, sensitive neurograms, and needle EMG were completely normal. The Mini-Mental Status Exam (MMSE) score was 10/30, and the routine CSF studies were normal. The electroencephalogram (EEG) showed moderate abnormalities. MRI of the proband and her second sister showed hyperintense streaking of the medial medullary lamina and mild cerebellar atrophy, which suggested MPAN (Fig. 1).

Her sister was a 26-year-old female, the second child of these related parents. She developed the first symptoms, including limb stiffness, gait impairment, posture incoordination, and occasional falls at the age of 18 years. She also began to complain of weakness in the lower extremities with rest tremors. At the age of 21 years, difficulties in swallowing and drinking, concentration disturbances, slow reactions, poor memory, and learning problems have developed. Forced crying and forced laughing was also presented occasionally. At 23 years of age, she was referred for neurologic evaluation in 2014. On admission, the physical exam revealed dystonia of the upper extremities, postural instability, wide-based gait, spastic tetraparesis with hyperreflexia and positive Babinski sign. However, mental disorders and autonomic symptoms were not present during the course of the disease. Ophthalmological investigation, EMG examination, and routine CSF studies were normal. The EEG showed moderate abnormalities. The MMSE score was 12/30. MRI of the proband and her second sister showed hyperintense streaking of the medial medullary lamina and mild cerebellar atrophy, which suggested MPAN(Fig. 1). All laboratory tests, including urinalysis, were normal.

**Fig. 2:**
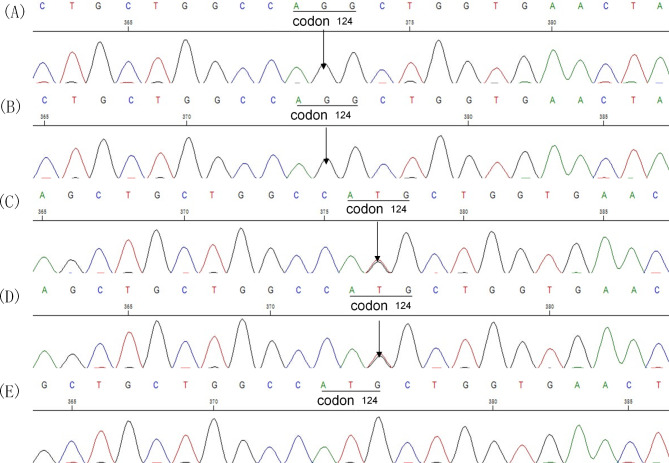
A and B show electrophoresis images of the proband and her second sister (exon 3: c.371T > G, p. Met124Arg, homozygous missense mutation). C and D show electrophoresis images of normal genes (heterozygous missense mutations) of the proband’s parents. The electrophoresis image of her third sister do not show sequence abnormalities (E)

**Fig. 3:**
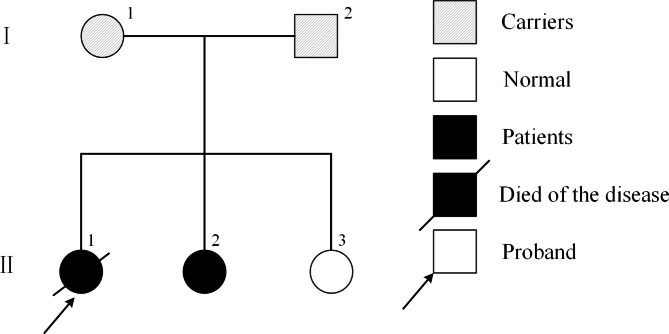
Family pedigree: Symbols: roman numbers to the left of the pedigrees denote generations. The arrow indicates the proband. Circles indicate females and squares indicate males. Fully black symbols indicate patients. Fully black with diagonal lines represents the patient’s death from the disease. Striped symbols denote the carriers

Whole-exome sequencing revealed a homozygous mutation in the *C19orf12* gene (exon 3: c.371T > G, p. Met124Arg) in the proband and the second sister, but not in the parents and the youngest sister (Fig. 2). This missense mutation indicated the diagnosis of MPAN with autosomal recessive inheritance (Fig. 3). The gene mutation site we found was located in the hot mutation location, and this mutation site was not reported in normal people, with a frequency of 0%. In addition, although the variant reported herein has not been previously reported, the substitution of methionine by other amino acids at the same site is confirmed to be a pathogenic missense mutation [9, 10]. According to the ACMG guidelines, the variants we found belong to the category “likely pathogenic variants” (PM1 + PM2 + PM5 + PP4) [11].

These two sisters were hospitalized again in 2017 because of the aggravation of symptoms. The proband had severe incontinence and edema of both lower limbs, and her second sister was unable to stand independently at that time. The proband and her second sister received levodopa and trihexyphenidyl to see if these therapies could provide benefit for the parkinsonism and dystonia, but there is no improvement of the symptoms after two weeks of treatment. Moreover, both of them developed obvious drowsiness. DFP, an iron chelator, was then experimentally prescribed to the proband and her second sister. The proband responded poorly to the iron removal treatment, and exhibited acute deterioration, including psychiatric disturbances and motor symptoms. She died from a severe pulmonary infection after two months of iron chelation therapy. The conditions of the second sister became stable after receiving the DFP treatment. She was followed up for four years, and at the latest follow-up (December 1, 2021), she reported that no new neurological deficit symptoms appeared after hospital discharge.

## Discussion

We presented two MPAN sisters with typical phenotypes and new gene site of mutation. The proband and her second sister also have similar clinical symptoms, and the two sisters both showed hypo-intensity in bilateral SN and GP on T(2)WI, FLAIR and SWI of MRI without the “eye-of-the-tiger” sign. These findings indicate that the main clinical symptoms, as well as the imaging findings of these patients with the novel mutation loci were similar with previously reported MPAN patients with other types of genetic mutation loci [12]. Optic atrophy, a typical symptom of MPAN for patients with early onset, was absent in our patients [13–15]. The second sister denied any vision loss during four years of follow-up. Although the two sisters suffered from the same gene mutation, the proband had faster disease progression. The condition in the proband rapidly switched to progressive deterioration after childbirth. *C19orf12* encodes a small transmembrane protein that is involved in the synthesis of free fatty acids and the expression of valine leucine and isoleucine biochemical in mitochondria to reduce the level of coenzyme A (CoA) [16, 17]. In MPAN, the changes in physiological hormones and lipid metabolism caused by pregnancy could also influence CoA metabolism, and thus accelerate the deterioration [18]. In addition, liver peptide hepcidin is a main regulator of systemic iron homeostasis, which controls serum iron to a relatively stable level by degrading iron transporters in enterocytes cells and macrophages [19]. Hepcidin expression is often inhibited by iron deficiency, expansion of erythropoiesis, anemia/hypoxia and so on. Obviously, gravida are more likely than normal people to suffer from the above-mentioned conditions to cause inhibition of the expression of the iron modulators, and finally to give rise to iron overload including brain tissue [20]. There were no studies that combined the gestation history to MPAN till now, further studies are required to verify the association. We also found that proband had refractory incontinence and severe mental symptoms at the end of the disease, but her sister did not show any of these symptoms during the course of the disease. Furthermore, fecal incontinence has never been reported in previous studies. This finding was consistent with a study from Russian, which reported that paraplegia and incontinence were symptoms indicative of disease deterioration [21]. Therefore, incontinence, especially fecal incontinence, maybe a symbol of the advanced stage of the disease.

To date, the therapeutic options for NBIA disorders remain largely thorny. Treatments including dopaminergic drugs, anticholinergics, tetrabenazine and even deep brain stimulation surgery is unsatisfactory and none of them cannot slow the disease progression [22]. Recently, the efficacy of DFP in iron removal for some PKAN patients has been gradually recognized and some studies shows that the earlier the disease, the more obvious the benefits [23–26]. DFP is also currently considered safe in the treatment of PKAN, with no serious adverse effects and no deaths due to the use of DFP in any patients. A study with 86 PKAN patients found that anemia was the most common adverse effect in the DFP treatment group compared to the placebo group, accounting for approximately 20% of adverse effects, but none of the patients discontinued treatment due to anemia. Only three patients discontinued DFP treatment because of moderate neutropenia [25]. DFP has also been used to treat Friedreich ataxia [27]. The researchers found that patients tolerated small doses of DFP well (on DFP 20 mg/kg/day), some patients showed a little decline in the left ventricular mass index, and only one patient experienced reversible neutropenia, but none developed agranulocytosis. However, more adverse events, such as exacerbating ataxia, occurred in excess of 40 mg/kg/day. This study shows that in addition to the importance of detecting blood cells and cardiac ultrasound during DFP treatment, it is essential to have the correct dose of medication. Unfortunately, there were few therapeutic studies on iron chelators in MPAN. A 13-year-old patient with a 2-year treatment of DFP remained clinically stable and showed slowly decreasing brain iron contents in the SN [28]. Another 35-years-old MPAN patient received DFP treatment for “nonsense talking”, but the therapy had to be discontinued due to gastrointestinal side effects. After the drug was stopped, he developed severe mental symptoms and incontinence soon [1]. Our study used DFP to treat two adult sisters with MPAN, but the therapeutic effects were different. The iron deposition was a little heavier and widely in the proband (Fig. 1I and K) than that in her second sister (Fig. 1M and O), and the proband also had heavier clinical symptoms. Previous studies had shown that iron deposition was associated with disease severity, suggesting that iron overload may not represent the initiating factor that triggers neurodegeneration but remains one of the major steps in the pathological cascade [29]. A recent study found extensive oxidative damage to lipids and proteins in neurons in 35-year-old MPAN patients’ biopsied cortical tissues indicating that iron overload likely contributed to oxidative brain damage [1]. In the MPAN cell model, the reduction of iron levels by DFP could also alleviate oxidative damage [30]. The findings of these studies provide a theoretical basis for the DFP treatment of MPAN. In addition, since the course of the proband’s disease was shorter than that of the second sister, it is also possible that the iron deposition rate in her brain tissue was faster than the second sister resulting in her neurological function not having time to compensate. It is worth paying attention to the proband showed initial symptoms at the age of 26, while the second sister had onset of disease at 18. A previous study also reported that MPAN in some late-onset adult patients progressed more rapidly than patients with early-onset [31]. We also do not rule out that worsening symptoms in proband patients may be influenced by a combination of age of onset, disease severity, and pregnancy. However, the second sister did not have any serious adverse reactions during 4 years of DFP use, and the four-year stable condition in the second sister suggests that early DFP treatment may be useful in preventing further deterioration of neural function.

## Conclusion

We report two sisters received DFP treatment of adult-onset MPAN. We want to highlight that DFP may have a beneficial effect in stabilizing symptoms in MPAN patients, especially in patients without severe autonomic dysfunction and cognitive mental impairment in the early disease stage. However, clinicians should be cautious given the potential deterioration in severely affected patients. We also hope that there will be more observational studies or relevant randomized controlled trials using DFP in different grades of patients to verify our results.

## Data Availability

All data generated or analysed during this study are included in this published article.

## Abbreviations

C19orf12: Open reading frame 12 on chromosome 19
MPAN: Mitochondrial membrane protein-associated neurodegeneration
NBIA: Neurodegeneration with Brain Iron Accumulation
PKAN: Pantothenase kinase-associated neurodegeneration
DFP: Deferiprone
CSF: Cerebrospinal fluid
SN: Substantia nigra
GP: Globus Pallidum
SN: Substantia Nigra. T(2)WI:T2-weighted imaging
FLAIR: Fluid attenuated inversion recovery sequence
SWI: Susceptibility weighted imaging
MRI: Magnetic resonance imaging
